# Comparison of three-fold converted hematocrit and micro-hematocrit in pregnant women

**DOI:** 10.1371/journal.pone.0220740

**Published:** 2019-08-01

**Authors:** Girum Tesfaye Kiya, Fitsum Mesfin Zewudie

**Affiliations:** 1 School of Medical Laboratory Sciences, Institute of Health, Jimma University, Jimma, Ethiopia; 2 Clinical Chemistry Laboratory Unit, Jimma Medical Center, Jimma University, Jimma, Ethiopia; Stony Brook University Health Sciences Center School of Medicine, UNITED STATES

## Abstract

**Background:**

Anemia is one of the common complications of pregnancy. Hemoglobin concentration, Hematocrit, and Red cell count are laboratory tests used to diagnose anemia. In are source poor setting, there is a practice of using three-fold converted Hematocrit. This study is designed to assess the association and acceptability of three-fold converted Hematocrit as compared to the standard Micro-hematocrit method, in pregnant women.

**Method:**

The cross-sectional study conducted from May 18 to June 12, 2018 involved 200 pregnant women who visited the Laboratory for a Hematocrit test. Three milliliter of venous blood was collected with EDTA tube to determine Hematocrit by the Micro-hematocrit method and Hemoglobin concentration measured by a HemoCue Hemoglobin B analyzer. A scatter plot, correlation coefficient, Bland and Altman plot, and Area under curve were employed to assess the agreement and acceptability of the calculated Hematocrit as compared to the standard Micro-hematocrit.

**Result:**

The correlation coefficient, Intraclass correlation coefficient and concordance correlation coefficient were 0.91, 0.94, and 0.89, respectively. The Bland and Altman plot showed a mean difference of 0.94 with the limit of agreement ranges from 0.6 to 1.3. The area under the receiver operating characteristics with cut-off point of Hematocrit <33% was 0.86. The sensitivity and specificity of the calculated method was 95.5% and 71.4%, respectively.

**Conclusion:**

Generally there is excellent association between the two methods. The two methods were identical within inherent imprecision of both methods. Hence, the Hematocrit value, threefold calculated from the Hemoglobin was found to be acceptable to diagnose anemia in pregnant women.

## Introduction

Iron deficiency anemia is one of the most common causes of anemia in pregnancy[1]. Anemia is defined as a Hemoglobin concentration <110g/l for pregnant women according to World Health Organization (WHO)[2].There are different laboratory tests that are employed to diagnose anemia such as Hematocrit, Hemoglobin, and Red cell count [1].

Hematocrit is a test that measures the percentage of blood that is comprised of red blood cells [3]. This is often referred to as packed cell volume (PCV) or erythrocyte volume fraction. A hematocrit value less than the reference interval is indicative of anemia and greater than the reference interval is indicative of polycythemia [4]. Micro-hematocrit method is a standard method for hematocrit determination according to Clinical and Laboratory Standards Institute (CLSI) [5]. A blood sample is filled three fourth in a capillary tube and centrifuged at specified revolution per minute (RPM) to find a separated compartments, of which, the ratio of the packed red cell at the bottom is measured. There is also conductivity method and automated Hematology analyzer to determine hematocrit value [6].

Hemoglobin is a protein in red blood cells which carries oxygen molecules to the tissues [7]. Hemoglobin measurement methods have shown progress starting from adding distilled water to measure the volume of blood as a hemoglobin level until its color matches with artificial comparator[8], to development of spectrophotometry and the hemoglobin cyanide (cynamethemoglobin) method, and then to automated hematology analyzer and point-of-care tests recently.

Hemocue HB 301 is a point of care test instrument which allows accurate determination of hemoglobin at the bedside and found to provide comparable result to other analyzers [9]. There is a disposable micro cuvette in it which has reagents necessary for both release of hemoglobin and conversion of it to a stable colored product for color intensity measurement [10].

In anemia diagnosis, hemoglobin gives direct measurement of oxygen carrying capacity while the hematocrit provides an indirect one [11]. Standard conversion has been used between these two tests (Hemoglobin = Hematocrit X3) as a cut-off point to estimate anemia [4].However, the conversion of hematocrit by Micro-hematocrit to hemoglobin has shown bias as compared to the direct measurement of hemoglobin, particularly in malaria endemic areas [12, 13]. This discrepancy might be due to the mean corpuscular volume (MCV) which is related to quantity of trapped plasma during centrifugation [14].The other way round conversion(Hematocrit = Hemoglobin x3) has not yet been evaluated for its comparability with the standard measurement which necessitates this study.

In are source poor setting where there is no automation and even in some Hospitals with automated Hematology analyzer, there is a practice of reporting hematocrit derived from hemoglobin as a three-fold conversion. Though the Micro-hematocrit is a standard method to measure hematocrit, it is time consuming and demands careful preparation and reading of the ratio. It’s use in under-resourced laboratories may also be limited as it demands a specialized centrifuge and a reliable supply of capillary tube [4]. In this case, Hemocue is a method mostly used to calculate hematocrit as it is easily portable and rapid test. Therefore, this study is aimed at determining the association and acceptability of a hematocrit value calculated from HemoCue HB 301as compared to the standard Micro-hematocrit method.

## Materials and methods

### Study setting

Facility based cross-sectional study was conducted in Jimma University Medical center (JUMC), Jimma, Southwest Ethiopia. The study was conducted from May 18 to June 12, 2018. A total of 200 pregnant women were involved in this study. All pregnant women who visited JUMC laboratory for Hematocrit test during the study period were included consecutively.

### Data collection and analysis

Socio-demographic and obstetric data were collected using structured questionnaire (S1 File). Three milliliter of blood sample was collected from each participant by a tube containing EDTA anticoagulant. A drop of blood from well mixed sample was used to fill the micro-cuvette of the HemoCue HB analyzer to determine Hemoglobin. The micro-cuvette was then inserted to the instrument which is factory pre-calibrated using Hemiglobincynanide (HiCN) standard and hence absorbance of the test solution is automatically converted to hemoglobin concentration and displayed in less than a minute[15].The hemoglobin concentration from Hemocue was converted to estimate Hematocrit, multiplying it by 3.Hematocrit was also measured by using manual Micro-hematocrit method. Three-fourth (75%) of capillary tube was filled, sealed on one side by a sealant and centrifuged at 12,000 RPM for 5 minutes to find layers of blood components. It is then placed on a Micro-hematocrit reader and the proportion of the bottom red pack was measured[16].Blood samples were measured by both methods within 2 hours of collection. A hematocrit value of less than 33% is used as a cut-off value to diagnose anemia in pregnant women.

### Quality assurance

All laboratory activities were performed by strictly following manufacturers’ instruction and specific standard operating procedures in the laboratory. All reagents and quality control materials used were checked for their expiry date and their functionality. All instruments and materials were calibrated before use. A daily optic check was performed using the red control cuvette when using the HemoCueHB301 analyzer. Both a high and a low liquid control were run each week to ensure functionality of the analyzer. Hematocrit results of both methods were recorded on standard report formats according to study participants’ unique identification number and attached to the respective questionnaire.

### Statistics

Descriptive statistics was used to determine frequency and percentage. The data were plotted from the calculated Hematocrit on the Y axis and Micro-hematocrit on the x axis to make visual assessment of the relation between the two methods. A correlation coefficient was analyzed to assess the association between the two methods. Interclass correlation coefficient and concordance correlation coefficient were also computed as an improved version to indicate the strength of association. Bland and Altman method was used to assess the agreement between the two methods which recommends the use of plots with bias and precision statistics and the plot consists of average of the two methods on X axis and the difference of the two methods on Yaxis [17–19]. The area under Receiver operating characteristics (ROC) was also determined with Hematocrit of less than 33% as a cut-off point, along with sensitivity and specificity.

### Ethics approval and consent to participate

Ethical clearance was obtained from the ethical review board of Faculty of health sciences, Jimma University. Permission was obtained from Medical director of JUMC and head of Laboratory of the Medical center. The purpose and importance of the study were explained and oral informed consent was taken from each participant. Confidentiality was maintained at all levels of the study.

## Results

A total of 200 pregnant women participated in this study. The majority (160(80%)) were within the age range of 20–34, of which 19 were anemic while 106(53%) were from rural area, of which 8 were anemic. Among the participants, 41(20.5%) attended either college or University whereas 31(15.5%) were illiterate. The estimated annual family income was 32.5% for the range of 10,000 to 15,000ETB. From the total pregnant women, 53% were on third trimester followed by 35% on 2^nd^ trimester and 12% in 1^st^trimester(Table 1).

**Table 1:** Socio-demographic data of study participants in JUMC; from May 15 to June 02, 2018.

Variables	Categories	Frequency	Percent (%)	Anemia^a^	
				Yes^b^	No^b^
Age	<20	19	9.5	0	19
	20–34	160	80.0	19	141
	35–49	21	10.5	1	20
Residence	Urban	94	47	12	82
	Rural	106	53	8	98
Educational status	Illiterate	31	15.5	3	28
	Primary 1–4	32	16	3	29
	Primary5-8	39	19.5	4	35
	Secondary	33	16.5	5	28
	Preparatory	24	12	1	23
	>12	41	20.5	4	37
Annually family income	<10.000	24	12	1	23
	10.000–15.000	65	32.5	8	57
	16.000–25.000	51	25.5	4	47
	>25.000	60	30	7	53
Gestational period	1^st^ trimester	24	12	2	22
	2^nd^ trimester	70	35	6	64
	3^rd^ trimester	106	53	12	94

^a^Hemoglobin level of less than 110g/L

^b^Frequency

The overall magnitude of anemia among study participants based on Hemoglobin measurement (Hb<11) by making use of Hemocue HB 301 was 20(10%) whilst by threefold converted Hematocrit (calculated), it was found to be 21(10.5%). This is also comparable when Hematocrit (Hct<33%) by Micro-hematocrit method is employed, which is 19(9.5%). Generally, 172(86%) pregnant women were within the normal range in both methods whilst there were 9(4.5%) women in Micro-hematocrit method and 7(3.5%) women in three fold converted method, with value above the upper limit.

**Fig 1:**
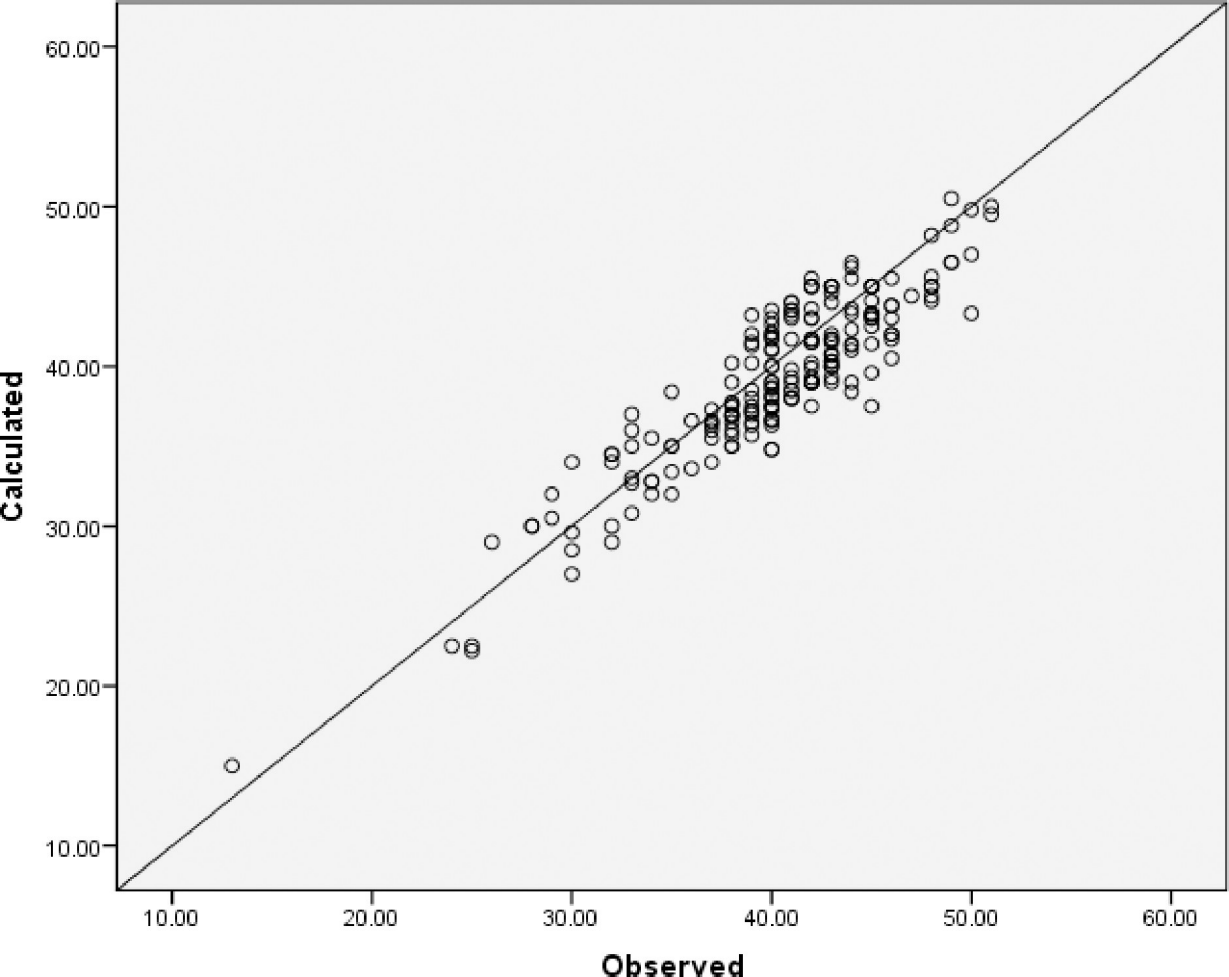
A scatter plot of the observed and calculated methods.

Data were plotted from Micro-hematocrit method (Observed) on the X-axis and three fold converted (calculated) on the Y-axis to make an overall visual assessment and the data were linear and distributed around the line; most of them within the reference range(Fig 1).

The correlation coefficient to measure the association between Micro-hematocrit method and three fold converted (calculated) method was = 0.91. The intraclass correlation coefficient was 0.94 while the concordance correlation coefficient was 0.89.

**Fig 2:**
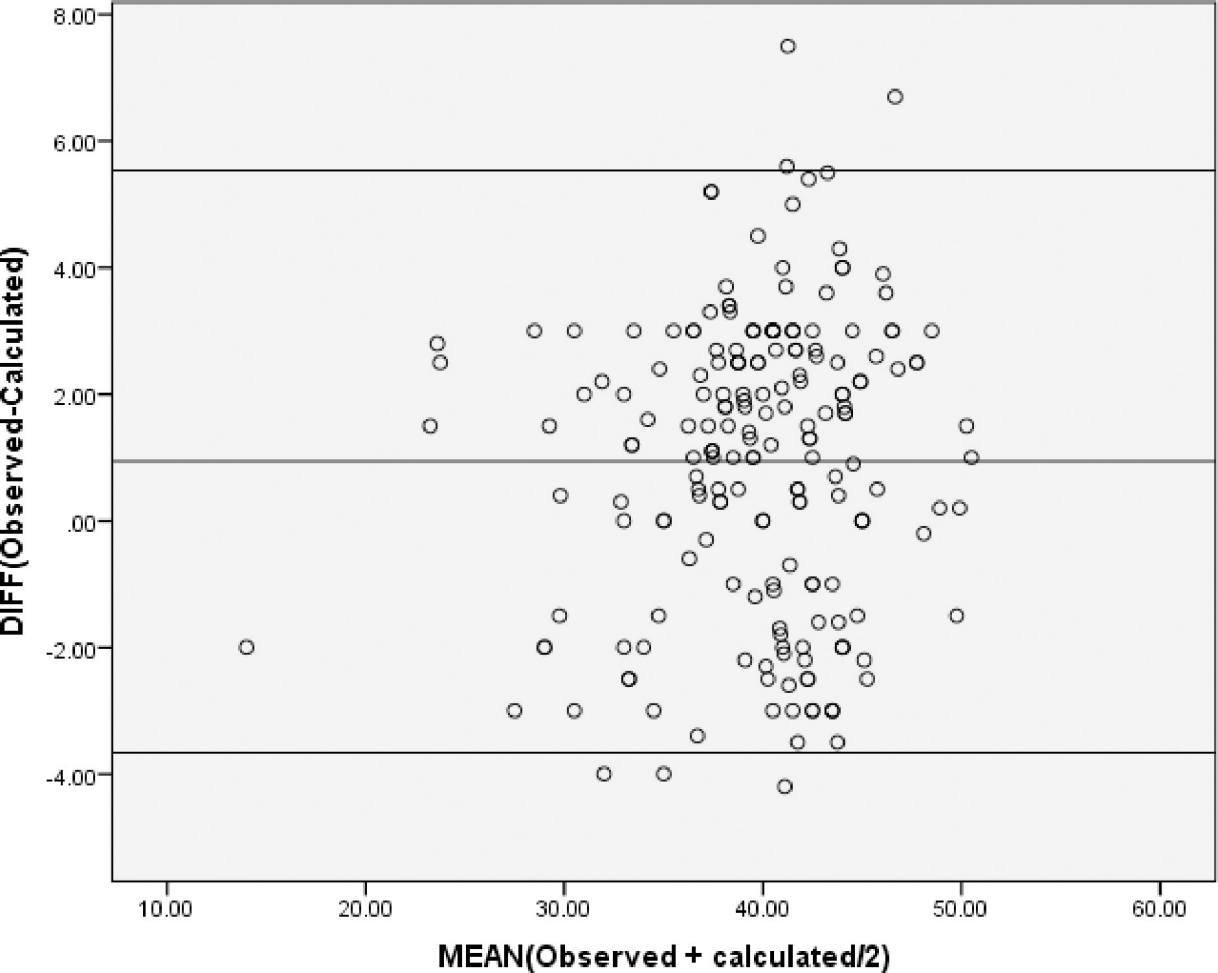
The Bland and Altman plot of limit of agreement.

The mean difference of measurements of the two methods based on Bland and Altman plot was 0.94 while the 95% limit of agreement ranges from 0.6 to 1.3(Fig 2).

**Fig 3:**
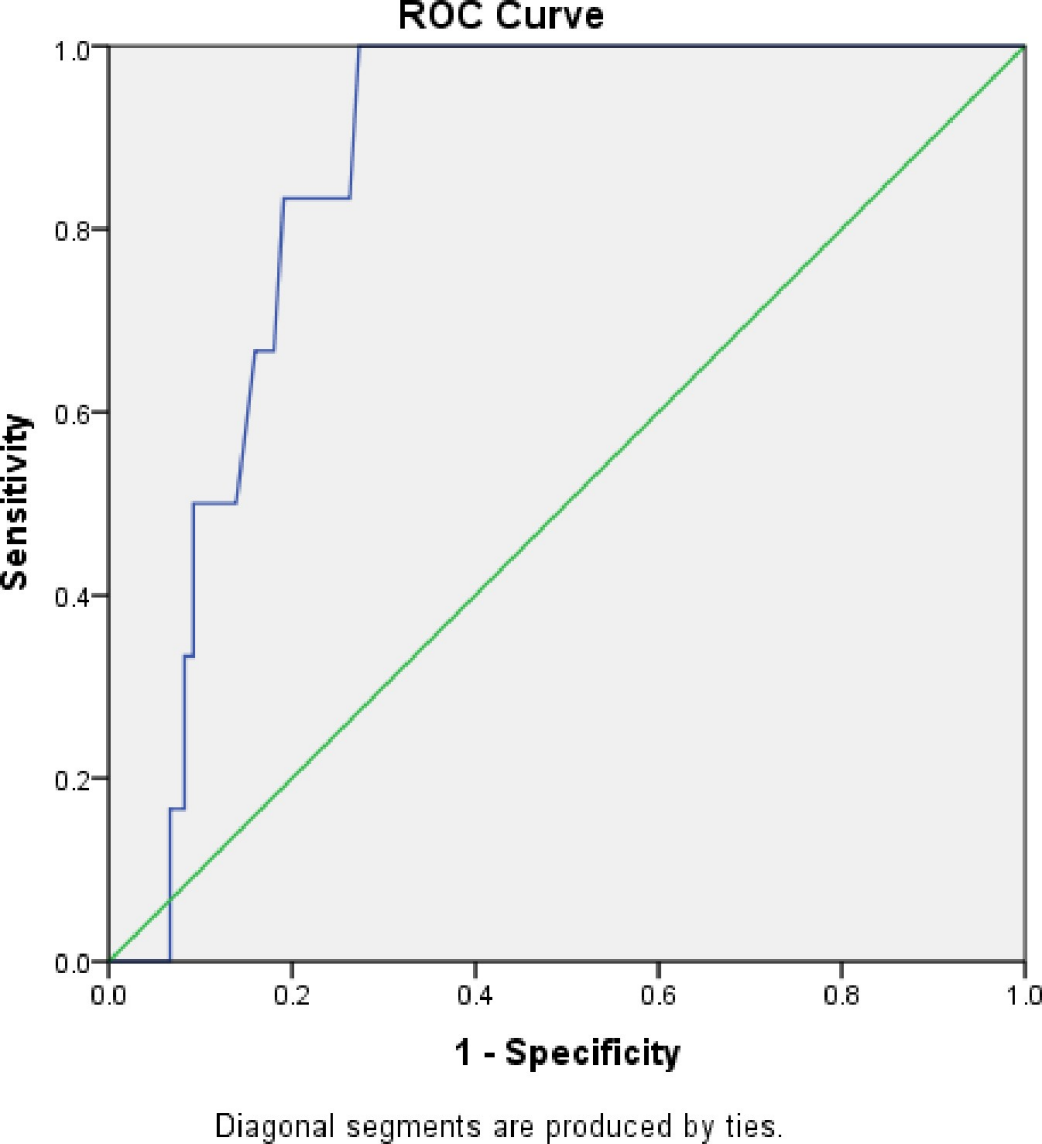
The area under the ROC curve.

The area under the ROC curve with a cut-off point of hematocrit less than 33% has shown that AUC = 0.86 with 95% confidence interval of 0.79 to 0.93 and p-value of 0.003(Fig 3). The sensitivity and specificity of the calculated method was also 95.5% and 71.4% respectively, taking Micro-hematocrit as a gold standard method of hematocrit determination.

## Discussion

A plot has shown that there is a linear association between the three-fold converted Hematocrit and Micro-hematocrit to measure Hematocrit in pregnant women. Furthermore, the correlation coefficient as well as the intraclass correlation coefficient and the concordance correlation coefficient showed that there is strong association between the two methods. It is important to note that while most of previous studies have shown association between observed hemoglobin and three fold converted hemoglobin, from hematocrit, this study is the other way round(hemoglobin to hematocrit). Previous studies has shown that the standard three fold conversion from hematocrit to hemoglobin underestimates the prevalence of low hemoglobin (<11g/dl) in children under five years of age in malaria endemic area [12]. It also underestimates the prevalence of anemia and low level hemoglobin in adults and children in malaria endemic area [20]. This might be due to trapped plasma during centrifugation which accounts for 3% in normal MCV, slightly higher value in macrocytic cells and a value as high as 5–6% in microcytic cells (14).

Another study in pregnant women has shown that hemoglobin from Drabkin’s method was significantly greater than three-fold converted hemoglobin which tends to be more prominent in anemic pregnant women (r = 0.5). It also suggested that there is no simple conversion factor between the two measurements [21]. Long interval between sample collection and analysis may be a reason for this discrepancy.

In the present study, Bland and Altman plot showed the acceptability (Mean difference = 0.94) of the calculated hematocrit as compared to the observed hematocrit seeing that the mean difference is not significantly different from zero. This indicates the two methods are Identical within inherent imprecision of both methods. A study in India showed that there was a correlation between calculated hemoglobin and observed hemoglobin (r = 0.94) but acceptability has failed based on the limit of agreement of the Bland and Altman method [22]. This might be due to the fact that the study only involved women on 3^rd^ trimester and the repeatability also gets worse as the severity of anemia increases.

The ROC curve is plotted to quantify how the new method can discriminate between anemic and non-anemic individuals. The area under the ROC curve which is 0.86 showed that the three-fold converted (calculated) method has a high predictive capacity to discriminate between anemic and non-anemic Individuals.

## Conclusion

Hematocrit calculated from Hemocue HB 301 Hemoglobin has acceptable agreement with Hematocrit determined by Micro-hematocrit method in pregnant women. Therefore, this study recommends that calculated Hematocrit as a three-fold conversion from Hemocue HB 301 Hemoglobin can be used to diagnose Anemia in pregnant women.

## Supporting information

S1 File Data collection tool for the study. (PDF)
