# Supplementary material for: Comparison of three-fold converted hematocrit and micro-hematocrit in pregnant women
Source: PLoS One. 2019 Aug 1;14(8):e0220740. doi: 10.1371/journal.pone.0220740 (PMC6675108; doi:10.1371/journal.pone.0220740)
Supplement: S1 File — (PDF) [file pone.0220740.s001.pdf]

**Jimma University**

**Institute of Health**

**Faculty of Health Science**

**School of Medical Laboratory Sciences**

**Data collection tool**

**Title**: Comparison of Three-Fold converted Hematocrit and Micro-hematocrit in Pregnant Women

**Part I: socio demographic Data**

1. Age in years ……………………..
2. Residence

   I.Urban -----------  II.Rural-----------
3. Educational status
   - I. Illiterate……….
   - II. primary (1-4)………
   - III. primary (5-8)………
   - IV. secondary (9-10)………….
   - V. preparatory (11-12)………….
   - VI. >12………..
4. Family income in ETB
   - I. <10,000
   - II. 10,000-15,000
   - III. 16,000-25,000
   - IV. >25,000
5. Gestational period
   - I. First trimester
   - II. Seconf trimester
   - III. Third trimester

**Part II. Laboratory investigation**

I. Hematocrit value by Hemocue analyzer ……………………

II. Hematocrit value by Micro-hematocrit method ………………

# ጅማ ዩኒቨርሲቲ ጤና ኢንስቲትዩት

# ጤና ሳንስ ፋካሊቲ

**መረጃ መሰብሰቢያ ቅጽ**

**የጥናቱ ርዕስ ፡- በሦስት እጅ የተባዛ ሔማቶክሪትን እና ማይክሮ ሔማቶክሪትን በነፍሰጡር ሴቶች ማወዳደር**

**ክፍል1፡- አጠቃላይ መረጃ**

1. ዕድሜ በዓመት ----------------------

2. መኖሪያ ስፍራ

   ሀ. ከተማ ለ. ገጠር

3. የት/ት ደረጃ

   ሀ. ያልተማረ

   ለ. ከ 1-4

   ሐ. ከ5-8

   መ. ከ9-10

   ሠ. ከ11-12

   ረ. ከ12 በላይ

4. ዓመታዊ ገቢ

   ሀ. ከ10. 000 በታች

   ለ. ከ10.000 - 15.000

   ሐ. ከ16.000 — 25.000

   መ. ከ25.000 በላይ

5. የእርግዝና ጊዜ

   ሀ. የመጀመሪያ ትራ ማስተር

   ለ. ሁለተኛ ትራይ ማስተር

   ሐ. ሶስተኛ ትራ ማስተር

ክፍል 2፡- የላቦራቶሪ ውጤት

ሀ. በሔሞኪው---------------

ለ. በ ማይክሮሔማቶክሪት ----------------------
